# Dynamic Expression of Novel MiRNA Candidates and MiRNA-34 Family Members in Early- to Mid-Gestational Fetal Keratinocytes Contributes to Scarless Wound Healing by Targeting the TGF-β Pathway

**DOI:** 10.1371/journal.pone.0126087

**Published:** 2015-05-15

**Authors:** Feng Zhao, Zhe Wang, Hongxin Lang, Xiaoyu Liu, Dianbao Zhang, Xiliang Wang, Tao Zhang, Rui Wang, Ping Shi, Xining Pang

**Affiliations:** 1 Department of Stem Cells and Regenerative Medicine, Key Laboratory of Cell Biology, Ministry of Public Health and Key Laboratory of Medical Cell Biology, Ministry of Education, China Medical University, 77 Puhe Street, Shenbei New District, Shenyang City 110013, Liaoning Province, China; 2 Department of Blood Transfusion, Shengjing Hospital of China Medical University, 39 Huaxiang Street, Tiexi District, Shenyang City 110004, Liaoning Province, China; 3 Department of General Practice, First Hospital of China Medical University, 155 North Nanjing Street, Heping District, Shenyang City 110001, Liaoning Province, China; Università degli Studi di Milano, ITALY

## Abstract

**Background:**

Early- to mid-gestational fetal mammalian skin wounds heal rapidly and without scarring. Keratinocytes (KCs) have been found to exert important effects on the regulation of fibroblasts. There may be significant differences of gestational fetal KCs at different ages. The advantages in early- to mid-gestational fetal KCs could lead to fetal scarless wound healing.

**Methods:**

KCs from six human fetal skin samples were divided into two groups: a mid-gestation group (less than 28 weeks of gestational age) and a late-gestation group (more than 28 weeks of gestational age). RNA extracted from KCs was used to prepare a library of small RNAs for next-generation sequencing (NGS). To uncover potential novel microRNA (miRNAs), the mirTools 2.0 web server was used to identify candidate novel human miRNAs from the NGS data. Other bioinformatical analyses were used to further validate the novel miRNAs. The expression levels of the miRNAs were further confirmed by real-time quantitative RT-PCR.

**Results:**

A total of 61.59 million reads were mapped to 1,170 known human miRNAs in miRBase. Among a total of 202 potential novel miRNAs uncovered, 106 candidates have a higher probability of being novel human miRNAs. A total of 110 miRNAs, including 22 novel miRNA candidates, were significantly differently expressed between mid- and late-gestational fetal KCs. Thirty-three differentially expressed miRNAs and miR-34 family members are correlated with the transforming growth factor-β (TGF-β) pathway.

**Conclusions:**

Taken together, our results provide compelling evidence supporting the existence of 106 novel miRNAs and the dynamic expression of miRNAs that extensively targets the TGF-β pathway at different gestational ages in fetal KCs. MiRNAs showing altered expression at different gestational ages in fetal KCs may contribute to scarless wound healing in early- to mid-gestational fetal KCs, and thus may be new targets for potential scar prevention and reduction therapies.

## Introduction

Scarless wound repair has been studied in numerous cases of fetal mammalian cutaneous wound healing. Early- to mid-gestational fetal mammalian skin wounds heal rapidly and without scarring and inflammation [1, 2, 3]. Late in gestation, fetal skin changes its response to injury from regeneration to the adult response of fibrosis, which leads to scar formation. In human skin, the transition occurs after approximately week 28 of gestation [1]. It is paramount to investigate the mechanism of scarless wound healing so as to translate it into clinical practice for both the development and application of novel therapeutic strategies against scar formation [4].

Regeneration may be the ideal way to restore tissue integrity and functional property upon injury [4, 5]. Many lines of evidence suggest that fibroblasts (FBs) play major roles in fetal cutaneous regeneration. FBs from fetal and adult skin are different in many respects, such as the ability to migrate, synthesis of collagen and hyaluronic acid [6–9], and the responses to inflammatory cytokine [10]. Altering the phenotype of FBs is the most studied approach for the controlling scar formation.

Not only FBs but also keratinocytes (KCs) may play a role in fetal cutaneous regeneration. KCs greatly affect the repairing process [11]. Re-epithelization stemming from KC proliferation is a crucial step for covering the denuded dermal surface during wound healing. Moreover, KC migration into wound tissue essentially occurs prior to cell proliferation within a few hours after wounding [12]. KCs have bi-directional interactions with FBs, particularly in wound healing [13], and have been shown to closely interact with FBs by regulating myofibroblast differentiation and stimulating FBs to synthesize growth factors which in turn will stimulate KC proliferation in a double paracrine manner [14]. There is also evidence showing that KC-FB interactions greatly enhance fetal cell secretion and accelerate scratch closure, indicating that fetal KCs may be a key player in scarless wound healing [15]. We hypothesize that there are significant differences between early- to mid- and late-gestational fetal KCs. The characteristics of late-gestational fetal KCs lead to scar formation.

MicroRNAs (MiRNAs) are a large family of highly conserved small non-coding RNAs that can play important regulatory roles in animals and plants by regulating a vast number of protein-coding genes [16]. They trigger translational repression and/or mRNA degradation mostly through binding complementarily to the 3'-untranslated regions (3'-UTR) of target mRNAs [17–19]. However, few studies have investigated the role of miRNAs in scarless wound healing, particularly in KCs. In this study, we investigate the differential expression of miRNAs between mid- and late-gestational fetal KCs to demonstrate the roles of miRNAs in KCs during the process of scarless wound healing.

## Materials and Methods

### 1. Fetal Skin Samples and Cells

KCs were obtained from six fetal skin samples. Full-thickness skin specimens from the lower legs of miscarried fetuses were divided into two groups: a mid-gestation group (gestational age 22–23 weeks, two males and one female) and a late-gestation group (gestational age 33–36 weeks, two males and one female). This study was approved by the Ethics Committee of Shengjing Hospital affiliated with China Medical University. Written informed consent was obtained from all of the patients before their participation.

A primary culture of KCs was prepared as previously described [20–22]. Briefly, full-thickness skin samples were incubated at 4°C overnight in Dispase II (Roche Applied Science, Indianapolis, IN, USA), and the dermal components were then removed through collagenase digestion. After 0.25% trypsin digestion, cultures of the released primary KCs from the epidermis were initiated using tissue culture flasks coated with collagen (Becton Dickinson Labware, Bedford, MA, USA) in Epilife growth medium (Invitrogen Ltd, Paisley, UK) supplemented with 1% human KC growth supplement (Invitrogen Ltd).

### 2. Construction of CDNA Libraries from Small RNA and Next-generation Sequencing (NGS)

The total RNA from the cells was extracted using the TRIzol reagent (Invitrogen, Carlsbad, CA, USA) according to the manufacturer’s instructions. For cDNA libraries construction and NGS, RNA samples were prepared using the Illumina TruSeq Small RNA Sample Preparation Kit according to the manufacturer’s instructions. The libraries were qualified using the Agilent 2100 High-Sensitivity DNA kit and quantified using the KAPA SYBR FAST qPCR Kit.

Cluster generation and sequencing on a Genome Analyzer (GA) (Illumina) IIx platform was performed following the manufacturer’s standard cBot and sequencing protocols. For multiplexing sequencing, 35 cycles of a single read were used to sequence the small RNAs. Image analysis and base calling were performed using the Illumina instrument software.

### 3. Analysis of Sequence Data

After adapter sequences were removed, the reads were aligned to the human genome of Ensembl using the Bowtie program [23] to filter out the reads in which the linker sequences were either mutated or absent. The high-confidence trimmed reads were then aligned to known miRNAs available in the miRBase (Release 18) in order to obtain sequences that either matched or did not match known miRNAs [24]. We screened the unmatched reads against a non-coding RNA database (Release 10) [25] to remove contamination from human non-coding RNAs, such as snRNAs, snoRNAs, rRNAs and tRNAs. Using the rest reads, the mirTools 2.0 web server [26] was used to identify novel miRNA candidates. For human miRNA prediction, we chose miRDeep 2.0 [27, 28] to run the web server and then filter out the sequence reads with a frequency of less than 10 counts. The pre-miRNA sequences were mapped to the human genome (human Genome v19, UCSC Genome Browser) in order to identify reads with perfect matches. The minimum free energies and the secondary structures of the potential precursors were assessed using the Vienna RNAfold web server (http://rna.tbi.univie.ac.at/). MiRNAs were considered to be conserved if at least 50% of the overall mature sequence was identical and the seed sequence (nucleotides 2–8) matched perfectly [29]. Potential target genes of known miRNAs were predicted using miRanda (http://www.miRNA.org/miRNA/home.do/). The gene 3’-UTRs were obtained from the Ensembl and miRanda databases. We considered at least one part of the 3’UTR of target mRNAs and the whole seed sequences of novel miRNA candidates were reverse-complemented perfectly with each other. The transforming growth factor-β (TGF-β) pathway was obtained from KEGG (http://www.genome.jp/kegg/). MiRNAs that showed significant correlation with the TGF-β pathway were considered to target not less than two primary genes of the pathway.

### 4. Small RNA Preparation

The total RNA, inclusive of small RNAs, was extracted using the mir-Vana miRNA Isolation Kit (Ambion, Austin, TX, USA) according to the manufacturer’s instructions. The concentration and purity of RNA were controlled by ultraviolet spectrophotometry (A260/A280 >1.9) using a Thermo Scientific Nanodrop 2000c Spectrophotometers (Thermo, Wilmington, Delaware, USA). After the 3’-termini were polyadenylated by *Escherichia coli* poly(A) polymerase (E-PAP) at 37°C for 45 min using the Poly(A) Tailing Kit (Ambion) following the manufacturer’s instructions, RNA was extracted with phenol-chloroform and precipitated with ethanol.

### 5. Real-time qRT-PCR

Real-time qRT-PCR was used to confirm the expression level of miRNAs. Reverse transcription was performed using a Superscript III first-strand synthesis system from a RT-PCR kit (Invitrogen), and real-time qRT-PCR was performed on a 7500 Real-Time PCR system (Applied Biosystems, Foster City, CA, USA) supplied with analytical software, using an Express SYBR greener qPCR Supermix Universal Kit (Invitrogen) according to the manufacturer’s instructions. The PCR reactions used for the amplification of miRNAs were conducted at 95°C for 30 s, followed by 45 cycles of 95°C for 5 s and 60°C for 34 s. The U6 mRNA level, as an endogenous reference, was used for normalization. After the final cycle, a melting curve analysis was conducted within the range of 55 to 95°C. The expression levels of miRNAs in late-gestational fetal KCs relative to mid-gestational fetal KCs were calculated using the equation 2^–ΔΔCT^ in which ΔC_T_ = C_T_ miRNA—C_T_ U6 [30]. The value of the relative expression ratio less than 1.0 was considered as low expression in late-gestational fetal KCs relative to mid-gestational fetal KCs, while the others were considered as high expression. The primers used for RT-PCR are given in S1 Table.

### 6. Statistical Analysis

The relative expression of miRNAs detected by either NGS or qRT-PCR was submitted to analysis using Student’s t-test. P values less than 0.05 were considered statistically significant. The statistically significant miRNAs whose expression changed by more than 2.0-fold in KCs at different gestational ages were considered to be significantly differentially expressed. The results detected by qRT-PCR were tested through three separate experiments. The correlation of the miRNA relative expression levels detected by NGS and qRT-PCR were analyzed by correlation analysis. All of the statistical analyses were performed using the SPSS 16.0 computer software.

## Results

### 1. CDNA Libraries Construction and NGS

To understand the potential contributions of miRNAs to fetal mammalian cutaneous scarless wound healing, we prepared cDNA libraries from small RNA extracted from each KC sample and examined the miRNA expression changes using NGS with Solexa technology. The raw data have been deposited in Gene Expression Omnibus (GEO) with accession number GSE 65342. Among the total of 99,354,224 raw reads that were detected from the six samples, 85,252,341 (85.81%) were high-quality reads (≥ 18nt). After alignment to the human genome (GRCH38), the number of genome-aligned reads was 74,678,115 (87.60% of the high-quality reads). The number of sequence reads that correspond to known miRNAs was 61,587,749 (82.47% of the genome-matching reads), as was determined by perfect sequence matching to the database of known miRNAs (miRBase release 18) (S2 Table). After removing the matched non-coding RNAs (Release 10), 8,755,258 reads remained for further analyses.

### 2. Identification of Novel miRNA Candidates

To uncover potentially novel miRNAs, the reads were analyzed using the mirTools 2.0 web server, and the miRDeep 2.0 software was used to identify candidate novel human miRNAs from the NGS data [26–28]. The results revealed the existence of 202 novel miRNA candidates and 29 known miRNAs (S3 Table) that were not listed in miRBase release 18. Of the 202 potential novel miRNAs, 106 candidates were detected by at least 10 counts, by NGS, indicating that they have a high probability of being novel human miRNAs (S4 Table).

**Fig 1:**
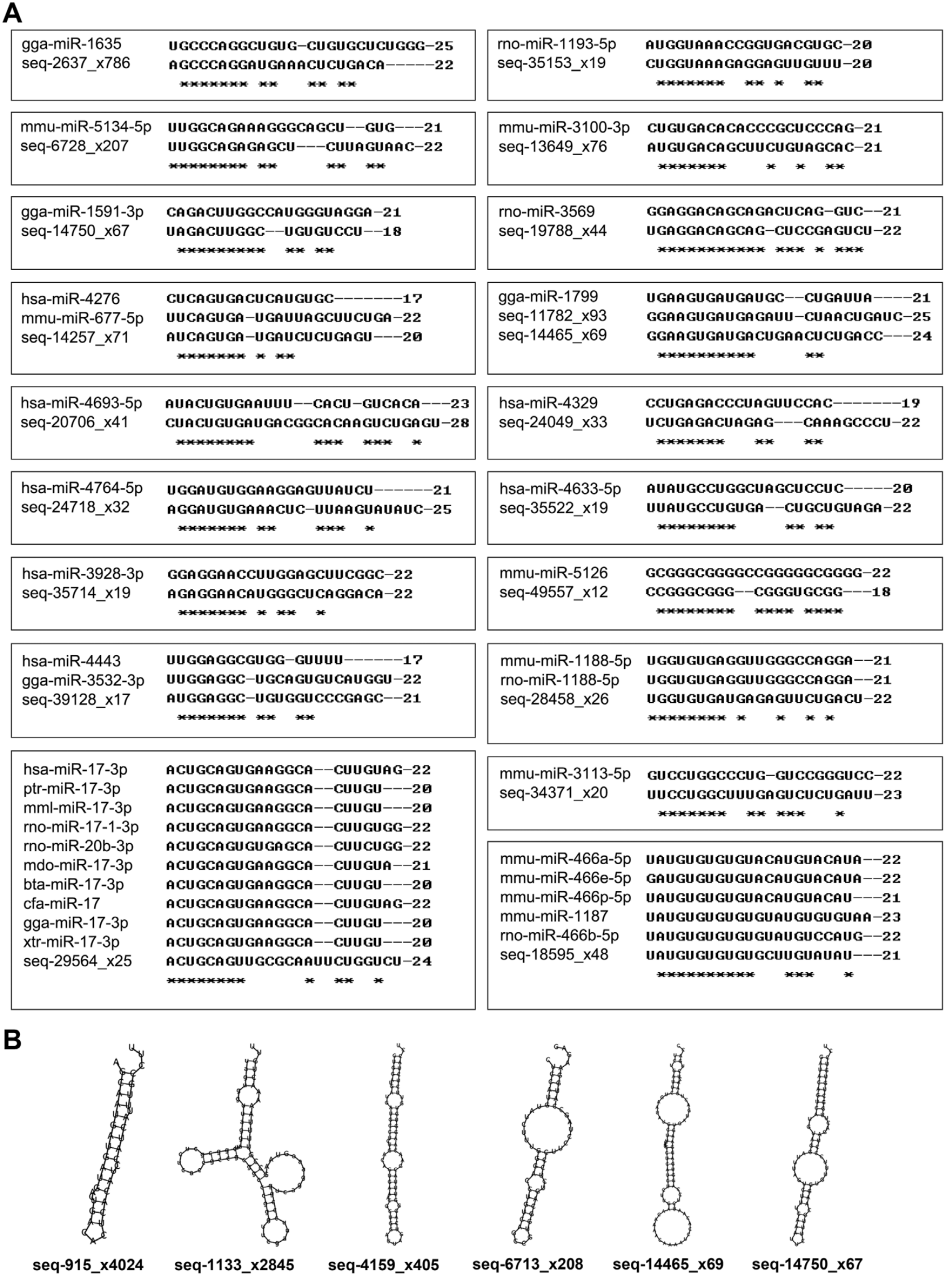
Novel miRNA candidates. (a) Sequence alignment of novel miRNA candidates with known miRNAs of other species. *: conserved nucleotide. bta: *Bos taurus*. cfa: *Canis familiaris*. gga: *Gallus gallus*. hsa: *Homo sapiens*. mdo: *Monodelphis domestica*. mml: *Macaca mulatta*. mmu: *Mus musculus*. ptr: *Pan troglodytes*. rno: *Rattus norvegicus*. xtr: *Xenopus tropicalis*. (b) Secondary structures of putative precursor hairpins corresponding to six novel miRNA candidates identified in this study. One (seq-6713_x208) of these novel miRNAs were found to be up-regulated in late-gestational fetal KCs, whereas the other five miRNAs were down-regulated (see also S7 Table).

Some of the novel miRNA candidates share seed sequences with known miRNAs in human and other species (S4 Table). Ten candidates (seq-3625_x495, seq-14257_x71, seq-20706_x41, seq-24049_x33, seq-24718_x32, seq-29564_x25, seq-35522_x19, seq-35714_x19, seq-39128_x17, and seq-40419_x16) share seed sequences with known *Homo sapiens* miRNAs (hsa-miR-4731-5p, hsa-miR-4276, hsa-miR-4693-5p, hsa-miR-4329, hsa-miR-4764-5p, hsa-miR-17-3p, hsa-miR-4633-5p, hsa-miR-3928, hsa-miR-4443, and hsa-miR-3128) respectively. Eight of these (seq-14257_x71, seq-20706_x41, seq-24049_x33, seq-24718_x32, seq-29564_x25, seq-35522_x19, seq-35714_x19, and seq-39128_x17) may become family members with the corresponding known human miRNAs because they are conserved miRNAs (Fig 1a). Interestingly, two novel miRNA candidates (seq-11782_x93 and seq-14465_x65) have the same seed sequences as gga-miR-1799, and their sequences were highly similar to each other (73.47% matched), but the alignment of the precursor sequence mapped to seq-11782_x93 in chromosome 9 rather than chromosome 5, where seq-14465_x65 resides. Moreover, the expression of seq-11782_x93 in late-gestational fetal KCs relative to that in mid-gestational fetal KCs was changed by 2.15-fold which was markedly higher than that found for seq-14465_x65 (0.26-fold). The mature sequence of one candidate novel miRNA, seq-18595_x48, was nearly identical to that of mmu-miR-466a/b/e/p-5p, particularly that of mmu-miR-466p-5p (three nucleotide differences; S4 Table and Fig 1a).

### 3. Validation of Novel miRNA Candidates

**Fig 2:**
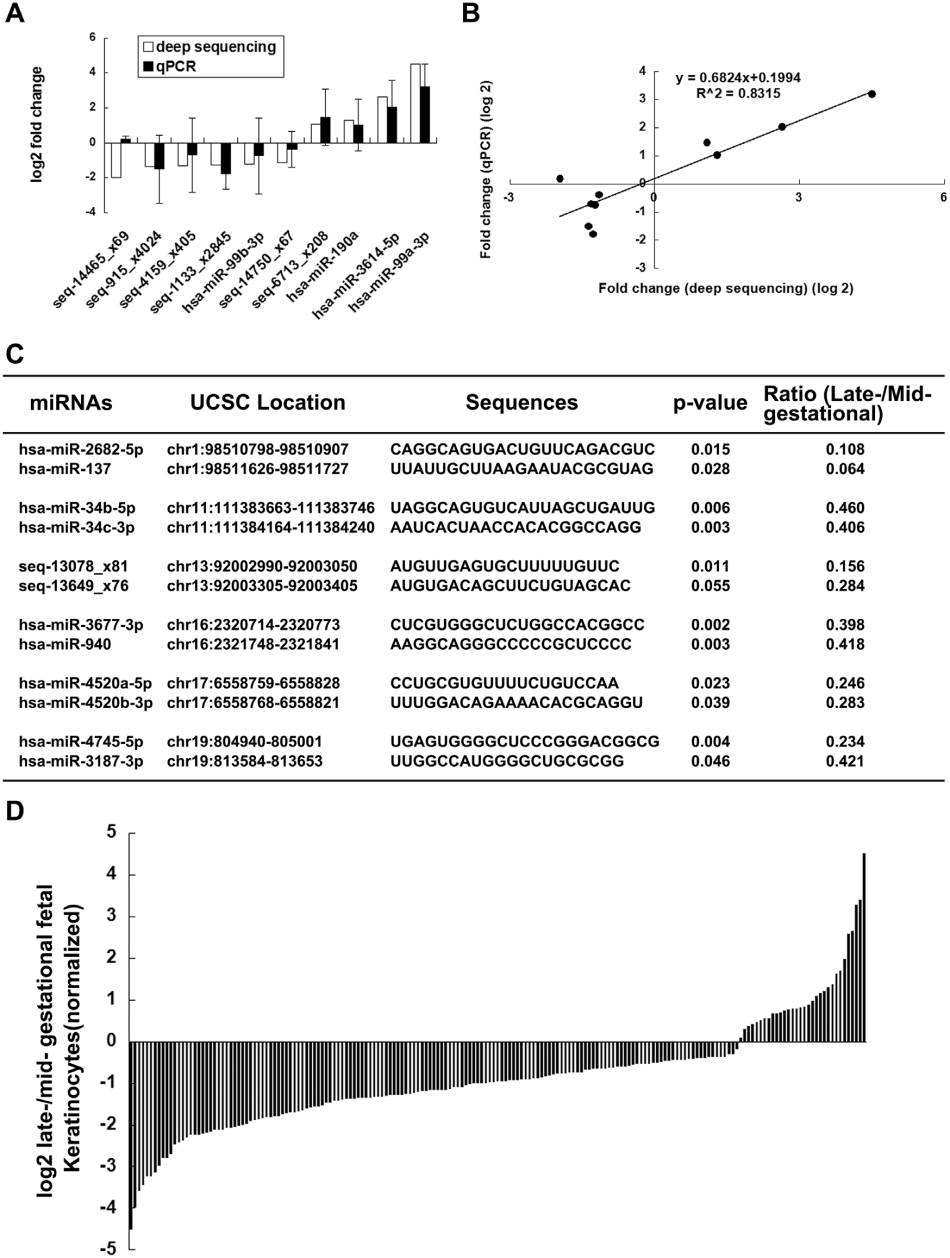
(a) Comparison of qRT-PCR data with NGS data for the six novel miRNA candidates {shown in (Fig 1b)} and four known miRNAs (hsa-miR-99b-3p, hsa-miR-190a, hsa-miR-3614-5p and hsa-miR-99a-3p). (b) Correlation analysis of the miRNA expression levels detected by NGS and qRT-PCR. The data were transformed to log2 values of the relative expression levels in late-gestational fetal KCs. The qRT-PCR results were normalized to the U6 snRNA expression levels. (c) Genomic locations of differentially expressed miRNAs and novel miRNA candidates found within 10 kb of each other. (d) Expression changes of statistically significant miRNAs in late-gestational fetal KCs detected by NGS. The data were transformed to log2 values of the relative expression levels in late-gestational fetal KCs.

The secondary hairpin structures and minimum free energies of the potential precursors were assessed using RNAfold, and the structures of the selected candidate novel miRNA precursors are shown in Fig 1b. To validate the expression of miRNAs detected by NGS, we tested the expression of six novel miRNA candidates and four known miRNAs via qRT-PCR and confirmed the expression of nine of them (Fig 2a). The correlation analysis showed that the miRNA expression detected by NGS exhibits good repeatability with that detected by qRT-PCR (Fig 2b).

We examined the genomic locations of novel miRNA candidates and known miRNAs that were found to be significantly differentially expressed in KCs at different gestational ages to determine whether that miRNAs found in particular genomic regions were potentially coexpressed and thus potentially coregulated. Twelve miRNAs from six groups composed of two miRNA each are located within close proximity (10kb) on chromosome 1, chromosome 11, chromosome 13, chromosome 16, chromosome 17 and chromosome 19 (Fig 2c). The expression of nearly all of these was significantly lower in late-gestational fetal KCs with the exception of seq-13649_x76 (ratio < 0.5, but p value > 0.05), suggesting that they may be coexpressed as miRNA clusters. Interestingly, six miRNAs (miR-17, miR-18a, miR-19a, miR-20a, miR-19b-1 and miR-92a-1) were found in close proximity to two novel miRNA candidates (seq-13078_x81 and seq-13649_x76) on chromosomes 13, even within 1kb. However, no statistically significance difference was found in the expression of the six miRNAs between mid- and late-gestational fetal KCs (S5 Table), indicating that the regulatory mechanisms of these miRNAs were complicated.

### 4. Dynamic Expression of miRNAs in Fetal KCs

**Fig 3:**
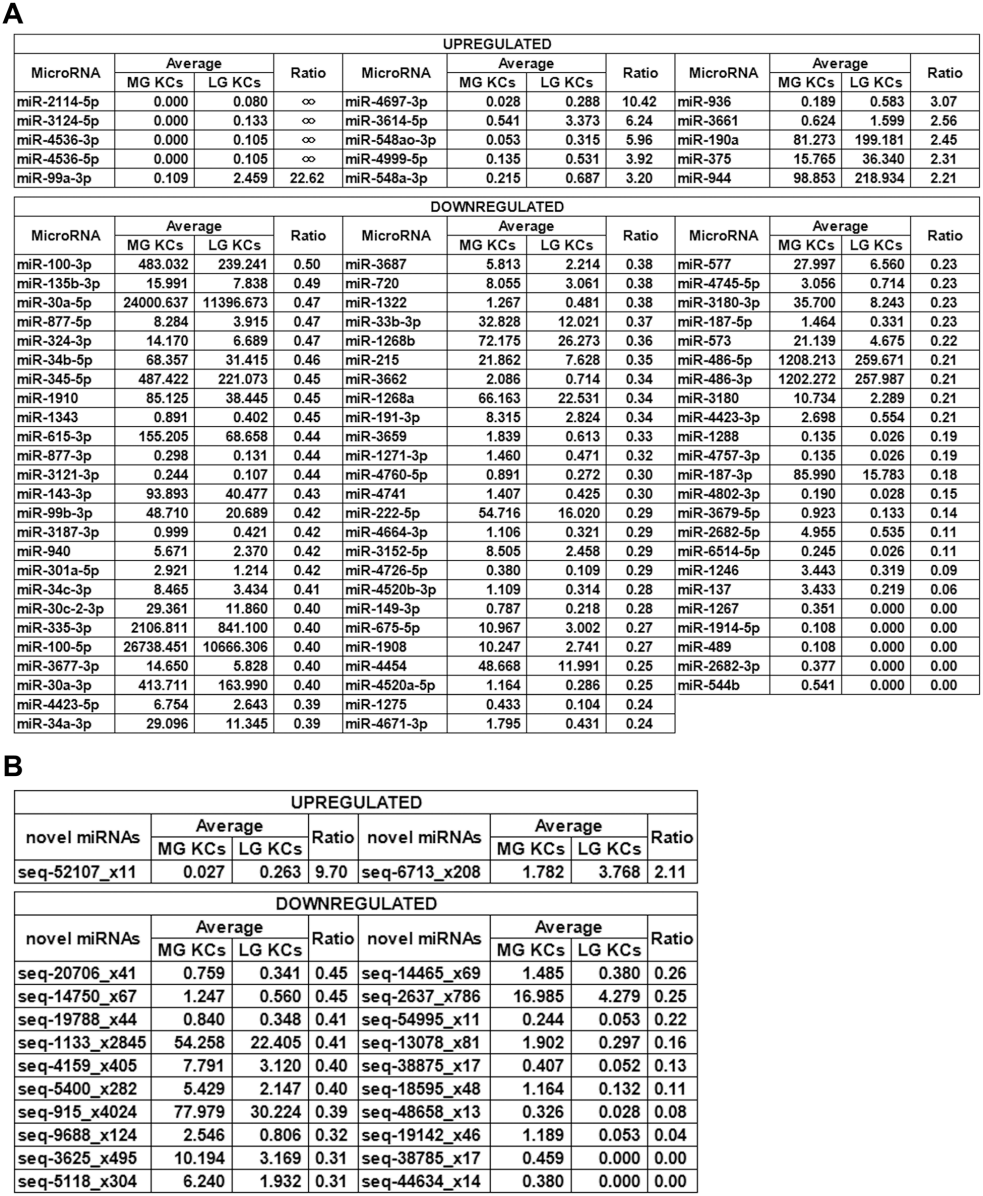
Significantly differentially expressed miRNAs between mid- and late-gestational fetal KCs. (a) Eighty-eight known miRNAs that exhibited a change in expression of more than 2.0-fold in late-gestational fetal KCs. Fifteen of these were statistically up-regulated, and the others were statistically down-regulated. The read number of the miRNAs was normalized to the total number of high-quality reads that matched the human genome from each sample (12591141/12266070/12065253/12074661/13021411/12659579). MG represents mid-gestational, and LG represents late-gestational. The ratio represents the relative expression of miRNAs in late-gestational KCs. (b) Twenty-two novel miRNA candidates that exhibited a change in expression of more than 2.0-fold in late-gestational fetal KCs. Two of them were statistically up-regulated, and the others were statistically down-regulated. The read number of miRNAs was normalized to the total number of high-quality reads that matched the human genome from each sample (12591141/12266070/12065253/12074661/13021411/12659579). MG represents mid-gestational, and LG represents late-gestational. The ratio represents the relative expression of miRNAs in late-gestational KCs.

We checked for perfect matches to known human miRNAs. Overall, 1170 known miRNAs were detected. To compare the expression levels of miRNAs between late- and mid-gestational fetal KCs, the read number of miRNAs was normalized to the total number of high-quality reads that were matched to the human genome (GRCH38) from each sample (12591141/12266070/12065253/12074661/13021411/12659579). The statistical results were analyzed using Student’s t-test. At different gestational ages, dynamic expression of miRNAs was observed in fetal KCs (Fig 2d) because 173 known miRNAs and 23 novel miRNA candidates were statistically significant (P values < 0.05, S6 and S7 Tables). The expression of 22 novel miRNA candidates and 88 known miRNAs was demonstrated to be significantly different because their relative expression was changed by more than 2.0-fold (known: 15 up-regulated and 73 down-regulated; novel: two up-regulated and 20 down-regulated) (Fig 3).

### 5. Differentially Expressed miRNAs Target the TGF-β Pathway

**Fig 4:**
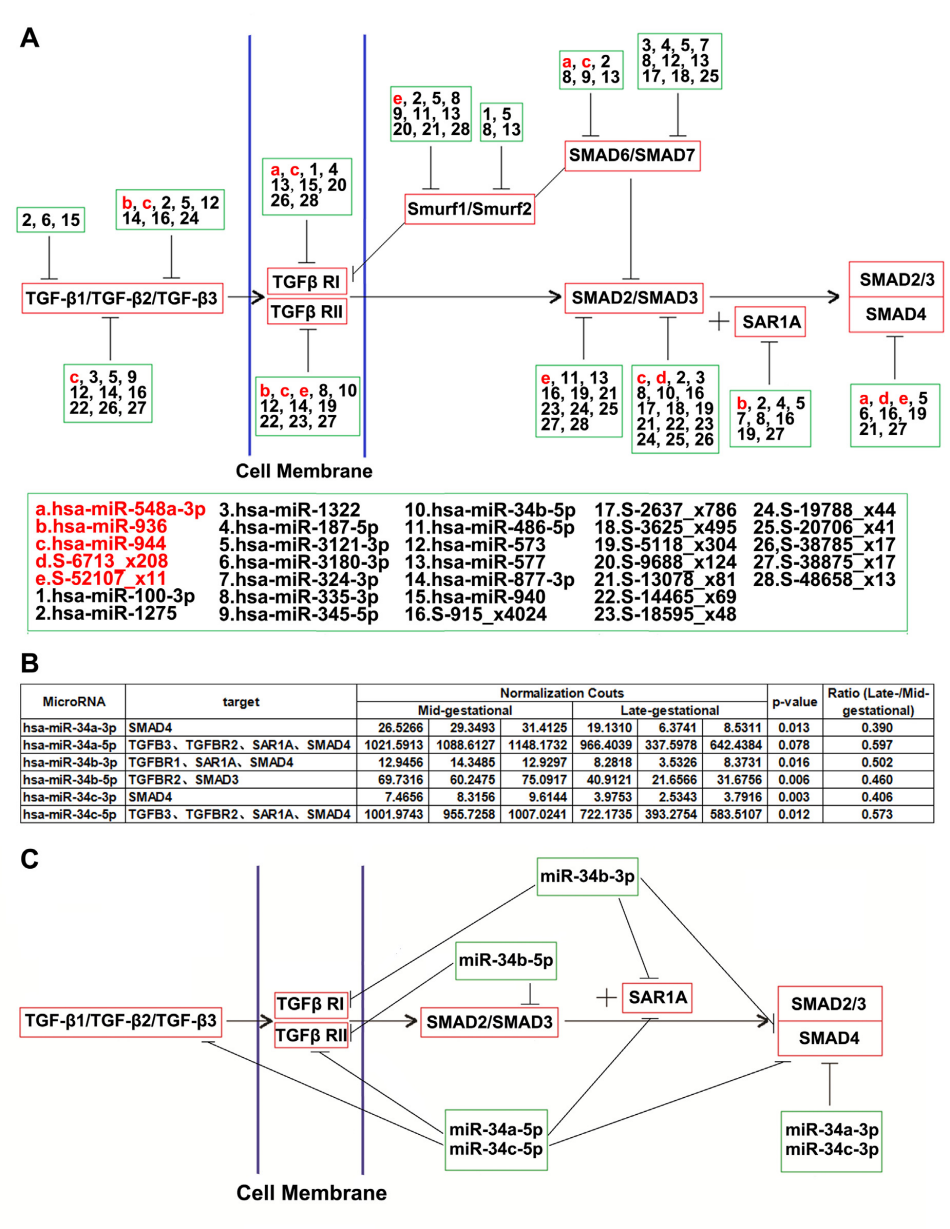
The TGF-β signaling pathway is predicted to be targeted by many significantly differentially expressed miRNAs. (a) Multiple miRNAs including novel miRNA candidates are predicted to target each of the genes implicated in the pathway, and each miRNA is predicted to target multiple genes in the pathway. The upregulated (> 2.0-fold) miRNAs are shown in red, and the downregulated (< 2.0-fold) miRNAs are shown in black. (b) MiRNA-34 family members are generally down-regulated in late-gestational fetal KCs. With the exception of miR-34a-5p, the expression levels of miR-34 family members in late-gestational fetal KCs were significantly lower (p value <0.05). MiR-34a-3p, miR-34b-5p, and miR-34c-3p changed by more than 2.0-fold. The read number of miRNAs was normalized to the total number of high-quality reads that matched the human genome from each sample. (c) MiRNA-34 family members suppress genes that are important members of the TGF-β pathway. TGF-β3, TGF-βRI, TGF-βRII, SMAD3, SMAD4 and SAR1A are involved in this regulation.

To further understand the relationship between miRNAs and fetal mammalian cutaneous scarless wound healing, we predicted the miRNA functions related to the TGF-β pathway. The downstream targets of the miRNAs that were significantly differentially expressed were further analyzed. MiRanda (http://www.miRNA.org/miRNA/home.do/) was used to predict the potential target genes of known miRNAs. We considered genes as potential targets of novel miRNA candidates if at least one part of their 3’UTR gene regions was reverse-complemented perfectly with the whole seed sequences of novel miRNA candidates. MiRNAs showed significant correlations with the TGF-β pathway if they targeted not less than two primary genes of the pathway. Under these conditions, we found that 33 miRNAs exhibit significant correlations with the TGF-β pathway (Fig 4a). Eighteen were known miRNAs (three up-regulated and 15 down-regulated), and the other 15 (68.18% of total novel miRNA candidates which were significantly differentially expressed) were novel miRNA candidates (two up-regulated and 13 down-regulated).

Among all of the TGF-β pathway-related miRNAs, five known miRNAs (miR-3180-3p, miR-34b-5p, miR-877-3p, miR-936, and miR-940) and 10 novel miRNA candidates (seq-915_x4024, seq-5118_x304, seq-6713_x208, seq-14465_x69, seq-18595_x48, seq-19788_x44, seq-38785_x17, seq-38875_x17, seq-48658_x13, and seq-52107_x11) target pathway members that positively regulate the pathway. These miRNAs appear to be suppressors that play critical roles in the regulation of the TGF-β pathway. The novel miRNA candidate seq-915_x4024 has the maximum number of targets, including SAR1A, SMAD2, SMAD3, SMAD4, TGF-β2 and TGF-β3. The total count of seq-915_x4024 (4024) was also the highest among all of the novel miRNA candidates. The differentially expressed miRNAs may play in a regulatory mechanism that is potentially upstream of the TGF-β pathway, and the roles of the novel miRNA candidates should not be ignored.

### 6. The miRNA-34 Family is Down-regulated in Late-gestational Fetal KCs and Extensively Targets the TGF-β Pathway

With the exception of miR-34a-5p (p value = 0.078), the expression levels of miR-34 family members in late-gestational fetal KCs were significantly lower than those in mid-gestational fetal KCs (Fig 4b). The expression levels of miR-34a-3p, miR-34b-5p, and miR-34c-3p were changed by more than 2.0-fold, suggesting that these miRNAs are expressed at significantly lower levels. We considered that the expression of miRNA-34 family members is generally down-regulated in late-gestational fetal KCs.

To predict potential target genes of miRNA-34 family members, we used the miRanda online software. The results showed that the miRNA-34 family may extensively suppress genes that play important roles in the TGF-β pathway, including TGF-β3, TGF-βRI, TGF-βRII, SMAD3, SMAD4 and SAR1A (Fig 4b and 4c). Both miR-34a-5p and miR-34c-5p, which have the same seed sequence, targeted TGF-β3, TGF-βRII, SMAD4 and SAR1A. The potential target gene of miR-34a-3p and miR-34c-3p was SMAD4. Although the mature sequences of miRNA-34 family members were highly similar to each others’, mature miR-34b does not have the same seed sequences as the others. The potential target genes of miR-34b-3p were TGF-βRI, SMAD4 and SAR1A. TGF-βRII and SMAD3 were potential target genes of miR-34b-5p.

## Discussion

Recently, rapid advances in next-generation sequencing (NGS) technologies allow miRNA detection at an unprecedented sensitivity [28, 31]. Since the application of NGS to miRNA detection, several analytical tools and databases have been developed to support miRNA data analyses [32]. These tools allow the analysis of a great number of miRNA-sequence data detected from high-throughput sequencing platforms for miRNA discovery across a broad spectrum of species [31]. For human miRNA discovery, MIReNA, miRDeep and mirTools are well used [26–28, 32–34]. MirTools 2.0 is a web service that provides annotation of ncRNA sequences and predicts novel miRNA candidates and potential known miRNA target genes based on NGS [26]. There are two programs for predicting novel miRNA candidates on the mirTools 2.0 web server: miRDeep 2.0 and mireap [26]. In this study, we detected small RNA expression in human fetal KCs using Illumina Solexa GA IIx, which is currently considered the most cost-efficient platform for miRNA sequencing studies [31]. After that, we performed the miRDeep 2.0 program which is well and widely used for novel human miRNA candidate prediction on the mirTools 2.0 web server [28, 32, 33, 35, 36] and found 106 novel miRNA candidates that are likely novel human miRNAs.

The role of miRNAs in scarless wound healing is only beginning to be uncovered. We detected the expression levels of miRNAs in fetal KCs at different gestational ages and found multiple miRNAs, particularly 22 novel miRNA candidates, that were significantly differentially expressed. We performed qRT-PCR to validate the expression levels of the miRNAs detected by NGS and the correlation analysis suggested that the results detected by NGS could almost represent the miRNA relative expression levels. Among all of the 110 significantly differentially expressed miRNAs, only 15.45% (15 known miRNAs and two novel miRNA candidates) were overexpressed. Our results show that many miRNAs, including several novel miRNA candidates, are significantly differentially expressed in fetal KCs during aging with a global downward trend in miRNA expression. The dynamic expression of miRNAs between mid- and late-gestational fetal KCs suggests that the differential expression of miRNAs in fetal KCs may be involved in the process of fetal mammalian cutaneous wound healing. MiRNAs may play important roles by suppressing the expression of a series of mRNAs and even some key signal pathways in scarless wound healing.

Members of the TGF-β signal pathway play important roles during embryonal development and are involved in a variety of cellular effects, such as growth, differentiation, apoptosis, extracellular matrix synthesis, and cell migration [37]. The TGF-β pathway is also an important regulatory factor involved in multiple other physiological processes, including scarless wound healing [1, 38]. In all stages of wound healing, TGF-β is involved, and prolonged TGF-β activation is associated with fibrosis [37, 39, 40]. Because the expression of TGF-β was observed in the epidermis and no TGF-β-producing cells were found in the dermis, KCs seem to be the main source of TGF-β in healthy fetal skin [1, 39, 41]. KCs leading to excessive activation of the TGF-β signal pathway may be a key step that leads to excessive scar formation in mammalian cutaneous wound healing. In this study, we predicted potential target genes of miRNAs and found that 33 significantly differentially expressed miRNAs target at least two primary genes of the TGF-β pathway. Because these are significantly correlated to the TGF-β pathway, we considered them TGF-β pathway-related miRNAs. These differentially expressed miRNAs in fetal KCs may play key roles in the process of scar formation by targeting the TGF-β signal pathway.

At different gestational ages, 22 novel miRNA candidates were found to be significantly differentially expressed in fetal KCs. Nearly 70% (15 novel miRNA candidates) of these were TGF-β pathway-related miRNAs, indicating that the novel miRNA candidates found in this study were significantly associated with the TGF-β pathway. With the exception of two novel miRNA candidates (seq-6713_x208 and seq-52107_x11), all of the other miRNAs exhibited significantly lower expression in late-gestational fetal KCs. We hypothesized that the novel miRNA candidates that presented low expression in late-gestational fetal KCs lose their regulation to TGF-β signal pathway and thereby contribute to scar formation.

The human miR-34 miRNA precursor family consists of three members encoded by two different transcripts: miR-34a, which is encoded by its own transcript, and miR-34b and miR-34c, which share a common primary transcript. From each precursor miRNA, two mature sequences are excised from the 5' or 3' arm of the hairpin. Therefore, there are six mature miR-34 miRNAs: miR-34a-3p, miR-34a-5p, miR-34b-3p and miR-34b-5p, miR-34c-3p and miR-34c-5p. Of all the miR-34 family members, miR-34a has been well studied. It is known as a key suppressor of a series of tumors, including colorectal cancer, non-small-cell lung cancer, breast cancer, and pancreatic cancer [42–46]. Researchers have recently noted that there are relationships between miR-34a and tissue fibrosis [47–49].

In this study, we demonstrated that the expression of miR-34 family members is generally down-regulated in late-gestational fetal KCs by NGS. Furthermore, we predicted that miR-34 family members may extensively suppress the TGF-β signal pathway. These results were similar to the findings that miR-34a can suppress the expression of TGF-β and SMAD4 [50, 51]. We considered that the overexpression of miR-34 family members may contribute to scarless wound healing in mid-gestational fetal KCs by targeting the TGF-β pathway. However, the regulatory effects between miR-34 family members and TGF-β signal pathway are markedly more complicated. TGF-β may play a direct role in regulating miR-34a expression [52]. The overexpression of miR-34 family members may suppress the TGF-β signal pathway and leads to a loss of control of miR-34a. Furthermore, the mature sequences of miR-34 are highly similar to each other. Nucleotides 2–9 from the sequences of miR-34a-5p and miR-34c-5p are matched perfectly. These two miRNAs share the same seed sequences and may both target TGF-β3, TGF-βRII, SMAD4 and SAR1A. Nucleotides 2–10 from the sequence of miR-34b-5p are perfectly matched to nucleotides 1–9 from the sequence of miR-34c-5p. Although the potential target genes of miR-34b-5p are TGF-βRII and SMAD3, as predicted by miRanda, miR-34b-5p may have regulatory effects on the miR-34c-5p potential target genes TGF-β3, TGF-βRII, SMAD4 and SAR1A. Nucleotides 1–10 from the sequence of miR-34c-3p and nucleotides 2–11 from the sequence of miR-34b-3p are matched perfectly. It is possible that TGF-βRI and SAR1A can be targeted by both of these miRNAs.

In conclusion, we found 106 candidates that have a high probability of being novel human miRNAs in fetal KCs. Our study showed that the dynamic expression of miRNAs at different gestational ages in fetal KCs. Moreover, the significantly differentially expressed miRNAs, including some novel miRNA candidates and miR-34 family members, extensively target the TGF-β pathway. The overexpression of the novel miRNA candidates and miRNA-34 family members in early- to mid-gestational fetal KCs may contribute to scarless wound healing by targeting the TGF-β pathway.

## Supporting Information

S1 Table Real-time qRT-PCR Primers used for Amplification of the Expression Levels of MiRNAs. (DOC)

S2 Table All 1141 known human miRNAs expressed in fetal KCs matched to miRBase release 18. (XLS)

S3 Table Twenty-nine known human miRNAs that are not listed in miRBase release 18 detected by mirTools 2.0. (XLS)

S4 Table One-hundred-and-six novel miRNA candidates expressed in fetal KCs. The read number of miRNAs was normalized to the total number of high-quality reads that matched the human genome from each sample (12591141/12266070/12065253/12074661/13021411/12659579). The color red indicates a greater-than-2.0-fold increase in late-gestational fetal KCs. The color green indicates a greater-than-2.0-fold decrease in late-gestational fetal KCs. Seed sequence conservation: novel miRNA candidates share seed sequences with known miRNAs in human and other species. bta: *Bos taurus*. cfa: *Canis familiaris*. gga: *Gallus gallus*. hsa: *Homo sapiens*. mdo: *Monodelphis domestica*. mml: *Macaca mulatta*. mmu: *Mus musculus*. ptr: *Pan troglodytes*. rno: *Rattus norvegicus*. xtr: *Xenopus tropicalis*.
(XLS)

S5 Table MiRNAs located within close proximity (10 kb) to the two novel miRNA candidates (seq-13078_x81 and seq-13649_x76) on chromosomes 13, (XLS)

S6 Table One-hundred-and-sev enty-three statistically significant known miRNA expressed in fetal KCs. The color red indicates a greater-than-2.0-fold increase in late-gestational fetal KCs. The color green indicates a greater-than-2.0-fold decrease in late-gestational fetal KCs.
(XLS)

S7 Table Twenty-three statistically significant novel miRNA candidates expressed in fetal KCs. The color red indicates a greater-than-2.0-fold increase in late-gestational fetal KCs. The color green indicates a greater-than-2.0-fold decrease in late-gestational fetal KCs.
(XLS)
