# Supplementary material for: Dynamic Expression of Novel MiRNA Candidates and MiRNA-34 Family Members in Early- to Mid-Gestational Fetal Keratinocytes Contributes to Scarless Wound Healing by Targeting the TGF-β Pathway
Source: PLoS One. 2015 May 15;10(5):e0126087. doi: 10.1371/journal.pone.0126087 (PMC4433274; doi:10.1371/journal.pone.0126087)
Supplement: S1 Table — (DOC) [file pone.0126087.s001.doc]

Supplementary Table 1. Real-time qRT-PCR Primers for Amplification of Expression levels of MiRNAs

|  | Primer Sequence (5′-3′) |
| --- | --- |
| RT-primer-1 | GCTGTCAACGATACGCTACGTAACGGCATGACAGTGTTTTTTTTTTTTTTTTTTTTTTTTA |
| RT-primer-2 | GCTGTCAACGATACGCTACGTAACGGCATGACAGTGTTTTTTTTTTTTTTTTTTTTTTTTG |
| RT-primer-3 | GCTGTCAACGATACGCTACGTAACGGCATGACAGTGTTTTTTTTTTTTTTTTTTTTTTTTC |
| seq-14465_x69-F^a^ | GGAAGTGATGACTGAACTCTGACC |
| seq-915_x4024-F^a^ | AGCAATGATGATGACTGACA |
| seq-4159_x405-F^a^ | GTGGATGATGATGCCTCTGACA |
| seq-1133_x2845-F^a^ | GTAAGCCGTTTTAAAAACTGTT |
| seq-14750_x67-F^a^ | TAGACTTGGCTGTGTCCT |
| seq-6713_x208-F^a^ | CTCCATGTATCTTTGGGACCTGTC |
| hsa-miR-99b-3p-F^a^ | CAAGCTCGTGTCTGTGGGTCCG |
| hsa-miR-190a-F^a^ | TGATATGTTTGATATATTAGGT |
| hsa-miR-3614-5p-F^a^ | CCACTTGGATCTGAAGGCTGCCC |
| hsa-miR-99a-3p-F^a^ | CAAGCTCGCTTCTATGGGTCTG |
| miRNA-Reverse^b^ | GCTGTCAACGATACGCTACGT |
| U6 RNA-F^a^ | CGCTTCGGCAGCACATATAC |
| U6 RNA-R^b^ | TTCACGAATTTGCGTGTCAT |

^a^ Forward primer

^b^ Reverse primer
